# Mycotic rhinitis in a Mutton Merino ewe

**DOI:** 10.4102/jsava.v88i0.1429

**Published:** 2017-01-24

**Authors:** Rhoda Leask, Johan Steyl

**Affiliations:** 1 Faculty of Veterinary Science, Department of Production Animal Studies, University of Pretoria, South Africa; 2 Faculty of Veterinary Science, Department of Paraclinical Sciences, University of Pretoria, South Africa

## Abstract

Although nasal masses are uncommon in sheep and may have several causes, including neoplasia and bacterial, fungal and viral infections, these lesions may lead to economic losses resulting from weight loss and even death. It is therefore important to differentiate between various categories of upper respiratory tract obstructions and lower respiratory tract infections. The correct aetiological diagnosis of obstructive masses is essential for appropriate treatment and management to be given or action to be taken. The presentation, clinical signs, treatment and pathology of a case of suspected mycotic rhinitis in a 6-year-old Mutton Merino ewe, are described.

## Introduction

Nasal masses have been recorded in many species and may be caused by bacteria (botryomycosis) (Thompson, Van der Lugt & Olivier-Carstens 2001), fungi (zygomycosis) (Ketterer et al. 1992; Riet-Correa et al. 2008), viruses (De las Heras et al. 2003; Ortίn et al. 2003; Taboada 2014) and neoplasms (Choi, Jeon & Park 2000; Delano, Mischler & Underwood 2002; Pérez et al. 2004; Rogers & Gould 1998). Such masses are rare in sheep or are seldom reported (Aitkin 2007; Bath & De Wet 2000; Kusiluka & Kambarage 1996; Rogers & Gould 1998; Scott 2007). Bacterial nasal masses caused by *Pseudomonas aeruginosa* have been reported in cattle (Thompson et al. 2001). Fungal nasal growths that have been reported in several species include conidiobolomycosis (Ketterer et al. 1992; Riet-Correa et al. 2008), phaeohyphomycosis (Taboada 2014), aspergillosis (Taboada 2014), cryptococcosis (Gusmão da Silva et al. 2010; Taboada 2014) and rhinosporidiosis (Taboada 2014). Penrith et al. (1994) also reported two separate cases involving *Bipolaris* sp. and *Drechslera* sp. in cattle. Of these, only conidiobolomycosis (Ketterer et al. 1992; Riet-Correa et al. 2008) and cryptococcosis (Gusmão da Silva et al. 2010) have been reported in sheep. Neoplasms, although rare in ruminants (Delano et al. 2002; Pérez et al. 2004), include ossifying fibromas (Choi et al. 2000; Rogers & Gould 1998), osteomas (Pérez et al. 2004) and retroviruses such as enzootic nasal tumour virus (Ortίn et al. 2003; Svara et al. 2006) and pulmonary adenocarcinoma virus (Taboada 2014).

It is important to distinguish between upper and lower respiratory tract diseases in order to identify the cause of the respiratory distress. Once an upper respiratory tract problem has been identified, it should be noted whether the problem is owing to myiasis (*Oestrus ovis*) (McKinnon et al. 1982) or a nasal mass. Clinical signs associated with nasal masses include coughing, apnoea or abnormal breathing patterns (Gusmão da Silva et al. 2010) and open-mouth breathing (McKinnon et al. 1982); a nasal discharge that may be serous (Marks Stowe et al. 2012; McKinnon et al. 1982; Taboada 2014), mucoid (McKinnon et al. 1982), muco-purulent (Gusmão da Silva et al. 2010; McKinnon et al. 1982; Taboada 2014) or haemorrhagic (Taboada 2014); sneezing (McKinnon et al. 1982; Taboada 2014); head shaking, nostril flaring (McKinnon et al. 1982); stridor (McKinnon et al. 1982; Taboada 2014; Thompson et al. 2001); slight facial distortion (Marks Stowe et al. 2012; McKinnon et al. 1982; Riet-Correa et al. 2008) or exophthalmia (McKinnon et al. 1982; Riet-Correa et al. 2008); anorexia (McKinnon et al. 1982) and weight loss (Taboada 2014). The location, size and growth rate determine the clinical signs seen (McKinnon et al. 1982).

Successful treatment for botryomycosis includes surgical excision, followed by antibiotic treatment (Thompson et al. 2001), whereas zygomycosis has been treated with a number of antifungal medications, including amphotericin B, fluconazole, and clotrimazole and itraconazole (Taboada 2014).

## Clinical case

A 6-year-old, 60 kg Mutton Merino ewe with a 1-week-old lamb at foot presented with unilateral mucoid nasal discharge on the left-hand side, mild epistaxis on the same side, inspiratory and expiratory dyspnoea and periods of open-mouthed breathing. A blood smear revealed mild neutrophilia, monocytosis, anisocytosis and polychromasia. A faecal flotation suggested a severe infestation with strongylids, presumably *Haemonchus contortus* (based on the fact that this sheep was from a summer rainfall area where haemonchosis has been diagnosed as a problem in this flock).

Radiographic examination of the chest demonstrated a moderate, diffuse bronchointerstitial pattern and mild oesophageal dilatation.

The ewe was treated with prednisolone 1% at 1 mg/kg (Prednisolone 10 mg/mL, VTech), florfenicol 20 mg/kg (extra-label) (Nuflor, MSD Animal Health), dexamethasone 2.5 mL (Kortico 2 mg/mL, Bayer Animal Health) and nebulised with bromhexine HCl solution 10 mg/5 mL (extra-label) (Bisolvon, Boehringer Ingelheim) and saline. The treatment was unsuccessful and the ewe was humanely euthanased by intravenous overdose of pentobarbitone (Eutha-naze, Bayer Animal Health).

**FIGURE 1:**
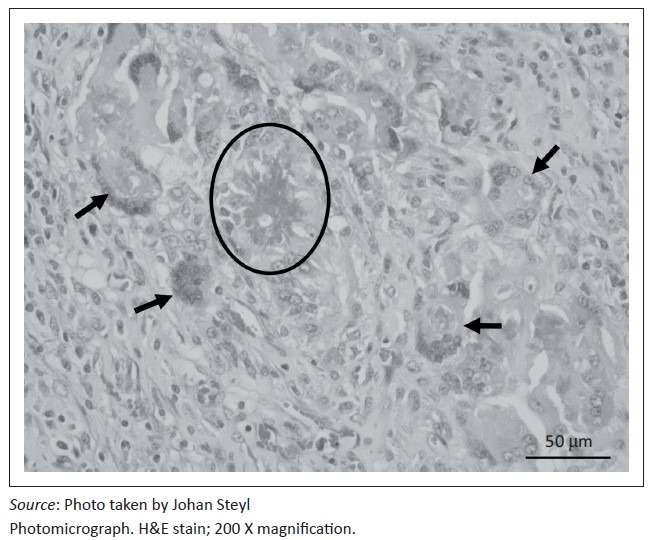
Hyper-eosinophilic Splendore-Hoeppli foreign material (circle) surrounded by large numbers of multinucleate giant macrophages (arrows) characteristic of the chronic inflammatory reaction associated with fungal infections.

**FIGURE 2:**
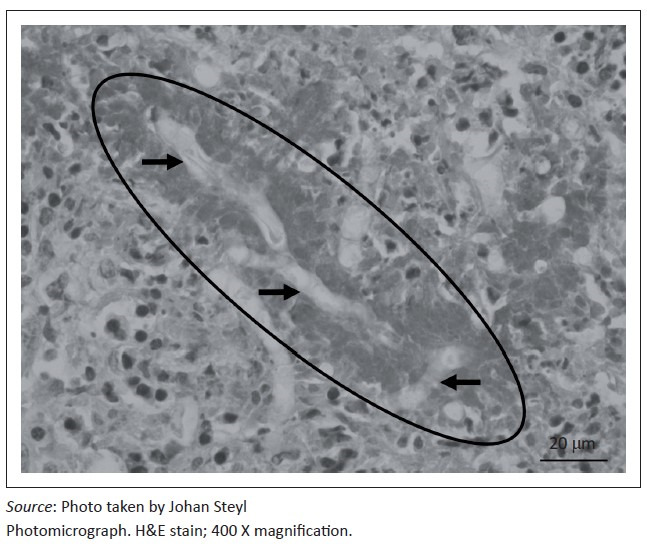
Hyper-eosinophilic Splendore-Hoeppli foreign material (circle) encasing ghost-like (negative H&E staining), pipe-shaped structures typical of fungal hyphae (arrows).

**FIGURE 3:**
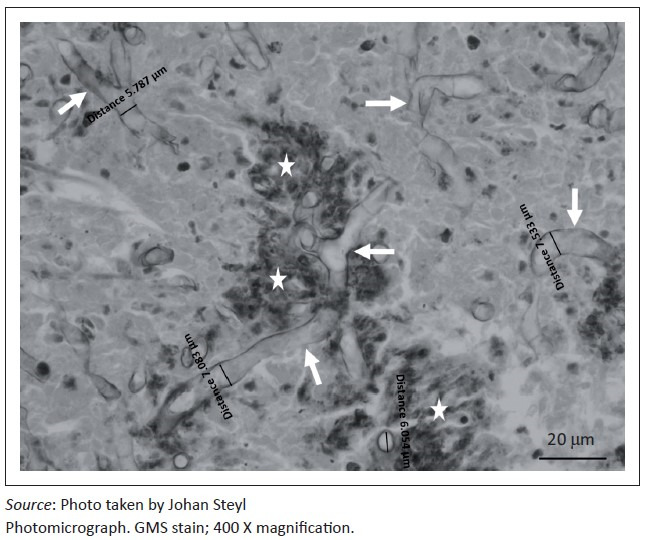
Non-septate, branching fungal hyphae (arrows) measuring between 5.7 μm and 7.5 μm in diameter among Splendore-Hoeppli foreign material (stars) and necrotic tissue debris.

At necropsy, a locally extensive, haemorrhagic, proliferative, invasive mass affecting the left nasal passage and extending from the ethmoid region to the ventral meatus was discovered. The mass also displaced the nasal septum significantly to the right and invaded its structure completely obscuring the nasal passages bilaterally to the nasopharynx. On examination of the other organs, the liver showed moderate fatty hepatosis. Samples of the mass and other organs were collected at necropsy in 10% buffered formalin (pH 6.5–6.8, CaCO_3_), processed and stained with haematoxylin and eosin (H & E) following standard methods (Bancroft & Gamble 2003). Microscopically, tissue of the nasal mass was characterised by multifocal to coalescing necrosis and severe, widespread pyogranulomatous inflammation surrounding radiating, club-shaped foreign substance (Splendore-Hoeppli material [Johnson 1976]) (Figure 1) occasionally containing large, hollow, pipe-like, poorly staining branching structures typical of fungal hyphae (Figure 2). The inflammatory component consisted of epithelioid macrophages, multinucleate giant cells, large numbers of degenerate neutrophils and fewer lymphocytes and plasma cells associated with extensive fibroplasia and scattered conchal osteolysis. In other regions, extensive haemorrhage, fibrin and neutrophilic exudation associated with granulomatous inflammation predominated. Special stains, namely Gommori’s methamine silver and Periodic Acid Schiff (Bancroft & Gamble 2003), for fungal organisms confirmed the presence of large, non-septate branching fungal hyphae central to and among the Splendore-Hoeppli and necrotic debris, respectively. The fungal hyphae were thin-walled and measured between 5.7 μm and 7.5 μm in diameter (Figure 3).

It is not known how long the lesion had been present in this ewe before it was large enough to cause respiratory distress. The predisposing factors in this case are also unknown.

## Discussion

For successful therapy, it is important to identify nasal masses as fungal, bacterial, viral or neoplastic as they present with similar clinical signs and may appear to be similar macroscopically. McKinnon et al. (1982) stated that there are several reasons as to why tumours of the nasal passages may be misdiagnosed, including early marketing for slaughter and the slow progression of clinical signs. Unless the cause is correctly identified, treatment may be largely unsuccessful or even detrimental, especially where antibiotics and corticosteroids are used initially on fungal growths (as was the case with this ewe and the ram reported by Gusmão da Silva et al. [2010]). Anecdotal evidence suggests that topical iodine may be successful in treating fungal granulomas, especially in cattle. In most cases of nasal masses, surgical excision followed by either systemic or topical remedies provides the best success rate (Thompson et al. 2001). Biopsies are useful in identifying the cause prior to treatment. Radiographs of the head can assist in distinguishing between pressure necrosis of bones or turbinates and proliferative bone growth (e.g. osteomas). There may also be a genetic component as far as neoplasms are concerned (Bath & De Wet 2000; Delano et al. 2002; McKinnon et al. 1982) and here careful selection and culling should be advised. In this case, fungal culture for definitive identification of the organism involved was not performed. However, the histomorphological characteristics of the fungus fall within the range of what is described for the fungal class of Zygomycetes. This group of organisms are environmental saprophytes that may cause disease in immunosuppressed individuals and/or upon inhalation of large numbers of spores (Quinn et al. 1994).
